# Patient Perception of Ideal Body Weight and the Effect of Body Mass Index

**DOI:** 10.1155/2014/491280

**Published:** 2014-12-29

**Authors:** Rozhin Naghshizadian, Amir A. Rahnemai-Azar, Kruthi Kella, Michael M. Weber, Marius L. Calin, Shahida Bibi, Daniel T. Farkas

**Affiliations:** Department of Surgery, Bronx-Lebanon Hospital Center, Albert Einstein College of Medicine, 1650 Selwyn Avenue, Suite 4E, Bronx, NY 10457, USA

## Abstract

*Objective*. Despite much effort, obesity remains a significant public health problem. One of the main contributing factors is patients' perception of their target ideal body weight. This study aimed to assess this perception. *Methods*. The study took place in an urban area, with the majority of participants in the study being Hispanic (65.7%) or African-American (28.0%). Patients presented to an outpatient clinic were surveyed regarding their ideal body weight and their ideal BMI calculated. Subsequently they were classified into different categories based on their actual measured BMI. Their responses for ideal BMI were compared. *Results*. In 254 surveys, mean measured BMI was 31.71 ± 8.01. Responses to ideal BMI had a range of 18.89–38.15 with a mean of 25.96 ± 3.25. Mean (±SD) ideal BMI for patients with a measured BMI of <18.5, 18.5–24.9, 25–29.9, and ≥30 was 20.14 ± 1.46, 23.11 ± 1.68, 25.69 ± 2.19, and 27.22 ± 3.31, respectively. These differences were highly significant (*P* < 0.001, ANOVA). *Conclusions*. Most patients had an inflated sense of their target ideal body weight. Patients with higher measured BMI had higher target numbers for their ideal BMI. Better education of patients is critical for obesity prevention programs.

## 1. Introduction

According to the World Health Organization (WHO), adults are considered overweight when their Body Mass Index (BMI) is 25 or higher and obese when their BMI is 30 or greater [1]. Recent data shows 62.8% of American adults to be overweight [2], with 35.7% obese [3].

Multiple strategies and programs oriented toward the prevention and treatment of the obesity have been initiated [4, 5]. These have been implemented at the different national [6], state [7], and local [8] levels. Despite all this, obesity continues to be an epidemic [9].

Lack of proper education and awareness plays a crucial role in the persistence of this epidemic. In order for many of these programs to be effective, it is essential for people to know if they are overweight. The first requirement for this is that they should have an accurate perception of their actual body weight. Many studies have investigated this topic and have found that people consistently overestimate their height and underestimate their weight, leading to an overall underestimate of their BMI [10, 11]. This fact is particularly true in heavier patients, as patients with higher BMI tend to underestimate their weight even more [12]. However, even if the patients are aware of their actual weight, a second prerequisite is to have a good understanding of one's ideal target weight. Several studies have looked at patients' perception of their weight status, but these have generally been done using subjective terms like “underweight,” “about right,” or “overweight” [13–17]. Some other surveys have used more descriptive terms like “body image” and “body satisfaction” [18–20]. Very few studies have asked subjects to give a numeric ideal weight for their height, thereby truly getting an accurate measure of where they think they stand relative to their ideal weight.

The purpose of this study was to accurately measure the patients' perception of their ideal target weight. In addition, the study sought to measure whether there were any differences between the respondents based on other factors. Of particular interest was if differences in measured BMI were associated with differences in perception of ideal body weight. In other words, did overweight and obese patients have different understandings of their healthy target weights compared to normal weight patients?

## 2. Methods

After obtaining Institutional Review Board approval, an anonymous survey was administered to patients in our outpatient clinic between July and December of 2011. Questionnaires were provided in both English and Spanish languages, and participation was voluntary. Patients were asked a few demographic questions, as well as whether they considered themselves underweight, of the right weight, or overweight. In addition, each patient was asked what he or she considered to be his or her “ideal body weight.” These were filled out in the waiting room, prior to the patients being seen by a nurse to record their height and weight. These measurements were recorded on their survey, and each respondent's “measured BMI” was calculated. Using the patients' response to ideal body weight, together with their actual measured height, each patient's perceived “ideal BMI” was calculated.

The patients were divided into four categories based on their measured BMI and the WHO classification: BMI < 18.5 (underweight), BMI 18.5–28.9 (normal), BMI 25–29.9 (overweight), BMI ≥ 30 (obese) [1]. The responses to ideal body weight (and calculated ideal BMI) were compared between the groups, using SPSS (Chicago, IL, USA) version 17.0 for statistical analysis.

## 3. Results

There were 254 valid survey responses during the study period. The demographic data is as shown in Table 1. 81.9% of patients were overweight and 52.8% were obese. The mean (±SD) measured BMI was 31.71 ± 8.02 (Table 1).

Patients' responses to their weight related perceptions, ideal body weight, and health habits are summarized in Table 2. 69.3% of the patients felt they were overweight. Most respondents (57.3%) admitted exercising less than most people, but the overwhelming majority (80.0%) felt they ate as healthy as or healthier than most people. The reason for the visit was related to weight in only 6.3% of patients (Table 2). The responses for ideal weight ranged from 100 to 250 pounds, with a mean of 153.22 ± 27.40 pounds. This corresponded with an ideal BMI that ranged from 18.89 to 38.15, with a mean of 25.96 ± 3.25. There were 153 respondents (60.2%) who answered with an ideal BMI greater than 25 (Table 2). Regarding their descriptive responses to their weight status, there was an appropriate shift between the groups, as summarized in Table 3. None of the underweight patients felt they were overweight, while 18.4% of the normal weight patients considered themselves overweight. In the overweight group this percentage was 69.9% and in the obese group this was 90.8%. Using a Pearson chi-square test, these differences were found to be highly significant, *P* < 0.001 (Table 3).

Ideal BMI varied widely between the different weight categories. Underweight patients felt their ideal BMI should be 20.14 ± 1.46, while normal weight, overweight, and obese patients answered their ideal BMI would be 23.11 ± 1.68, 25.69 ± 2.19, and 27.22 ± 3.31 respectively. In other words, the ideal BMI targets for obese patients (measured BMI ≥ 30) were higher than ideal BMI targets of overweight patients, which in turn were higher than the ideal BMI targets of normal weight patients. The responses of these various groups were compared using an ANOVA test and the differences were highly significant, *P* < 0.001 (Table 4). In subgroup analysis the females (151 females out of 229 total respondents) had lower responses for ideal BMI (25.61 ± 3.47) than the males (26.64 ± 2.74), with this difference being statistically significant (*P* = 0.023, ANOVA). When comparing between the various BMI groups, the differences between the groups was again highly significant in both the females and the males.

## 4. Discussion

The obesity epidemic is a major public health concern in the United States [9, 21]. According to recent studies, the estimated annual health care costs of obesity-related illness are incredible, $190.2 billion or nearly 21% of annual medical spending in the United States [22]. As a result, there are multiple national and local programs aimed at the prevention of obesity [4–9]. Yet despite all this, obesity continues to be a problem [3, 21]. Education plays a key role in all of these preventive programs. In order for these programs to be effective, it is essential that the targeted population understand if they are overweight. This requires that they not only be aware of their actual weight, but also have a good sense of what their ideal body weight should be. Previous studies have already demonstrated the lack of accuracy with patients' self-reported weight [10–12]. The focus of this study was to measure patients' insight to their target ideal weight. We found that, in our population, most patients had an inflated sense of what their ideal weight should be. In fact, the mean ideal BMI of all our patients was 25.96, which is actually in the overweight category. Over 60% of patients answered with an ideal weight that would put them in the overweight category.

When comparing responses between patients of different weights, we found that the higher the patient's measured BMI is, the higher they felt their ideal weight was supposed to be. Whereas normal weight patients responded with a reasonable mean ideal BMI of 23.11, the obese group of patients had a mean ideal BMI over 27. In other words, heavier patients had an increased target for their ideal weight, which meant they underestimated how overweight they were. This is obviously important when planning education and prevention programs. Many studies have demonstrated a strong association between perceived weight status and weight control behavior [23–30]. If patients do not realize how overweight they are, they are less likely to make any effort to change.

There have been some studies that looked at this issue in more subjective terms. Subjects were asked to describe themselves as being “the right weight,” “underweight,” or “overweight” [13–17]. Others have sought to divide these categories into narrower groups, using terms such as “slightly overweight” and “very overweight” [31]. Some have even measured this in other descriptive terms, measuring things like body image and body satisfaction [18–20]. Though there is value to this kind of a measurement, it is not very precise. Even in our own study, we did find that the obese patients were more likely to consider themselves overweight. However, it was only when we look at the actual numerical responses that we were able to see the true extent of the problem. Heavier patients understood they were overweight, but their increased perception of their ideal weight led to a lack of appreciation as to how overweight they were.

Our study has several limitations. For one, we used BMI as the main measure of weight status. There are well known problems with just using BMI, as it does not take into account muscle or fat content [32]. However, we do not think this was a major issue in our population, and BMI is certainly the standard used by most authorities, including the WHO [1]. A second limitation is that because of the urban setting of our hospital almost our entire population was either Hispanic or African-American. Since different ethnic groups have been shown to have different perceptions of weight [33], it is possible that the overall percentages and numerical responses of this study might not be generalizable to all patient groups. However, we feel that the difference seen between the BMI groups of patients is likely to persist in other patient populations as well.

Future areas of research could try to determine causation. Did patients first have an increased target weight and therefore allowed them to become obese as a result? Or did patients adjust their understandings of ideal weight at they got heavier? The latter seems more likely, but patients would have to be followed longitudinally in order to answer this question. Other areas to look at would be the effect of education. If we educate patients about their ideal BMI, would this lead to any changes in weight loss behavior? This is obviously the crucial question to answer if we want to implement new programs to prevent and treat obesity on a population level.

## 5. Conclusion

We found that most of our patients had a sense that they were overweight but also had an inflated perception of their target ideal body weight. This led to an underestimate of how overweight they actually were. We also found that obese and overweight patients had higher targets for their ideal body weight in comparison to normal weight patients. This difference in perception is something that needs to be addressed, if we are to have success in nutrition education and obesity prevention programs.

## Figures and Tables

**Table 1:** Demographics of survey respondents.

Gender	
Male	78 (30.7%)
Female	151 (59.4%)
Age	
<20	5 (2.0%)
20–29	22 (8.7%)
30–39	36 (14.2%)
40–49	64 (25.2%)
50–59	62 (24.4%)
60–69	37 (14.6%)
≥70	28 (11.0%)
Measured height (inches)	
Range	55–74
Mean	64.33
SD	3.886
Measured weight (pounds)	
Range	85–434
Mean	186.43
SD	51.125
Measured BMI (m/kg^2^)	
Range	16.06–61.51
Mean	31.71
SD	8.016
Measured BMI groups	
Underweight (BMI < 18.5)	6 (2.4%)
Normal weight (BMI 18.5–24.9)	40 (15.7%)
Overweight (BMI 25–29.9)	74 (29.1%)
Obese (BMI ≥ 30)	134 (52.8%)
Ethnicity/race	
Latino/Hispanic	167 (65.7%)
African-American/Black	71 (28.0%)
Other	9 (3.6%)
Multiple or invalid response	7 (2.8%)
Comorbidities	
DM	53 (20.9%)
HTN	125 (49.2%)
High cholesterol	65 (25.6%)
Heart disease	27 (10.6%)

**Table 2:** Weight related perceptions of respondents.

Do you consider yourself:	
Underweight	21 (8.3%)
The right weight	42 (16.5%)
A little overweight	95 (37.4%)
A lot overweight	81 (31.9%)
Not sure	8 (3.1%)
Missing, multiple, or invalid response	7 (2.8%)
How much do you exercise?	
Less than most people	146 (57.5%)
The same as most people	82 (32.3%)
More than most people	23 (9.1%)
Missing, multiple, or invalid response	3 (1.2%)
How healthy do you eat?	
Less healthy than most people	44 (17.3%)
The same as most people	149 (58.7%)
More healthy than most people	54 (21.3%)
Missing, multiple, or invalid response	7 (2.8%)
Is today's visit related to your weight?	
No	232 (91.3%)
Yes	16 (6.3%)
Missing, multiple, or invalid response	6 (2.4%)
Ideal body weight (pounds)	
Range	100–250
Mean	153.22
SD	27.406
Calculated ideal BMI (m/kg^2^)	
Range	18.89–38.15
Mean	25.96
SD	3.254

**Table 3:** Descriptive weight perception compared between the BMI groups (*P* < 0.001).

Actual BMI	Response				
	Underweight	The right weight	A little overweight	A lot overweight	Not sure
Underweight (BMI < 18.5)	5 (83.3%)	1 (16.7%)	0	0	0
Normal weight (BMI 18.5–24.9)	11 (28.9%)	18 (47.4%)	7 (18.4%)	0	2 (5.3%)
Overweight (BMI 25–29.9)	2 (2.7%)	18 (24.7%)	43 (58.9%)	8 (11.0%)	2 (2.7%)
Obese (BMI ≥ 30)	3 (2.3%)	5 (3.8%)	45 (34.6%)	73 (56.2%)	4 (3.1%)

Total	21 (8.5%)	42 (17.0%)	95 (38.5%)	81 (32.8%)	8 (3.2%)

**Table 4:** Ideal BMI responses compared between the BMI groups.

Measured BMI	*n*	Ideal BMI		
		Range	Mean	SD
Underweight (BMI < 18.5)	6 (2.4%)	18.89–23.03	20.14	1.467
Normal weight (BMI 18.5–24.9)	40 (15.7%)	20.12–27.43	23.11	1.681
Overweight (BMI 25–29.9)	74 (29.1%)	21.63–30.89	25.69	2.193
Obese (BMI ≥ 30)	134 (52.8%)	20.54–38.15	27.22	3.314

Total	254 (100%)	18.89–38.15	25.96	3.254

## References

[1] World Health Organization (2000). *Obesity: Preventing and Managing the Global Epidemic: Report of a WHO Consultation*.

[2] Gallup (2012). In U.S., majority overweight or obese in all 50 states. *Gallup Wellbeing*.

[3] Ogden C. L., Carroll M. D., Kit B. K., Flegal K. M. (2012). Prevalence of obesity in the United States, 2009-2010. *NCHS Data Brief*.

[4] Nihiser A. J., Lee S. M., Wechsler H. (2007). Body mass index measurement in schools. *Journal of School Health*.

[5] Roofe N. L. (2011). Improving families' nutrition knowledge through service learning. *Journal of Allied Health*.

[6] Wang Y. C., Orleans C. T., Gortmaker S. L. (2012). Reaching the healthy people goals for reducing childhood obesity: closing the energy gap. *The American Journal of Preventive Medicine*.

[7] Lake A. M. (2012). Pediatric obesity: preventive measures in early childhood. *Journal of Parenteral and Enteral Nutrition*.

[8] Stamatakis K. A., Leatherdale S. T., Marx C. M., Yan Y., Colditz G. A., Brownson R. C. (2012). Where is obesity prevention on the map? distribution and predictors of local health department prevention activities in relation to county-level obesity prevalence in the United States. *Journal of Public Health Management and Practice*.

[9] Mitchell N. S., Catenacci V. A., Wyatt H. R., Hill J. O. (2011). Obesity: overview of an epidemic. *Psychiatric Clinics of North America*.

[10] Gorber S. C., Tremblay M., Moher D., Gorber B. (2007). A comparison of direct vs. self-report measures for assessing height, weight and body mass index: a systematic review. *Obesity Reviews*.

[11] McAdams M. A., Van Dam R. M., Hu F. B. (2007). Comparison of self-reported and measured BMI as correlates of disease markers in US adults. *Obesity*.

[12] Villanueva E. V. (2001). The validity of self-reported weight in US adults: a population based cross-sectional study. *BMC Public Health*.

[13] Chang V. W., Christakis N. A. (2003). Self-perception of weight appropriateness in the United States. *The American Journal of Preventive Medicine*.

[14] Duncan D. T., Wolin K. Y., Scharoun-Lee M., Ding E. L., Warner E. T., Bennett G. G. (2011). Does perception equal reality? Weight misperception in relation to weight-related attitudes and behaviors among overweight and obese US adults. *The International Journal of Behavioral Nutrition and Physical Activity*.

[15] Yaemsiri S., Slining M. M., Agarwal S. K. (2011). Perceived weight status, overweight diagnosis, and weight control among US adults: the NHANES 2003–2008 Study. *International Journal of Obesity*.

[16] Chang V. W., Christakis N. A. (2001). Extent and determinants of discrepancy between self-evaluations of weight status and clinical standards. *Journal of General Internal Medicine*.

[17] Burke M. A., Heiland F. W., Nadler C. M. (2010). From “overweight” to “about right”: evidence of a generational shift in body weight norms. *Obesity*.

[18] Adami G. F., Gandolfo P., Campostano A., Meneghelli A., Ravera G., Scopinaro N. (1998). Body image and body weight in obese patients. *International Journal of Eating Disorders*.

[19] Docteur A., Urdapilleta I., Defrance C., Raison J. (2010). Body perception and satisfaction in obese, severely obese, and normal weight female patients. *Obesity*.

[20] Schwartz M. B., Brownell K. D. (2004). Obesity and body image. *Body Image*.

[21] Catenacci V. A., Hill J. O., Wyatt H. R. (2009). The obesity epidemic. *Clinics in Chest Medicine*.

[22] Cawley J., Meyerhoefer C. (2012). The medical care costs of obesity: an instrumental variables approach. *Journal of Health Economics*.

[23] Stephenson M. G., Levy A. S., Sass N. L., McGarvey W. E. (1987). 1985 NHIS findings: nutrition knowledge and baseline data for the weight-loss objectives. *Public Health Reports*.

[24] Kruger J., Galuska D. A., Serdula M. K., Jones D. A. (2004). Attempting to lose weight: specific practices among U.S. Adults. *American Journal of Preventive Medicine*.

[25] Bish C. L., Blanck H. M., Serdula M. K., Marcus M., Kohl H. W., Khan L. K. (2005). Diet and physical activity behaviors among Americans trying to lose weight: 2000 behavioral risk factor surveillance system. *Obesity Research*.

[26] Forman M. R., Trowbridge F. L., Gentry E. M., Marks J. S., Hogelin G. C. (1986). Overweight adults in the United States: the behavioral risk factor surveys. *The American Journal of Clinical Nutrition*.

[27] Riley N. M., Bild D. E., Cooper L. (1998). Relation of self-image to body size and weight loss attempts in black women: the CARDIA study. *The American Journal of Epidemiology*.

[28] Lynch E., Liu K., Wei G. S., Spring B., Kiefe C., Greenland P. (2009). The relation between body size perception and change in body mass index over 13 years. *The American Journal of Epidemiology*.

[29] Serdula M. K., Collins M. E., Williamson D. F., Anda R. F., Pamuk E., Byers T. E. (1993). Weight control practices of U.S. adolescents and adults. *Annals of Internal Medicine*.

[30] Dorsey R. R., Eberhardt M. S., Ogden C. L. (2010). Racial and ethnic differences in weight management behavior by weight perception status. *Ethnicity & Disease*.

[31] Gregory C. O., Blanck H. M., Gillespie C., Michele Maynard L., Serdula M. K. (2008). Health perceptions and demographic characteristics associated with underassessment of body weight. *Obesity*.

[32] Goacher P. J., Lambert R., Moffatt P. G. (2012). Can weight-related health risk be more accurately assessed by BMI, or by gender specific calculations of Percentage Body Fatness?. *Medical Hypotheses*.

[33] Gillum R. F., Sempos C. T. (2005). Ethnic variation in validity of classification of overweight and obesity using self-reported weight and height in American women and men: the Third National Health and Nutrition Examination Survey. *Nutrition Journal*.

